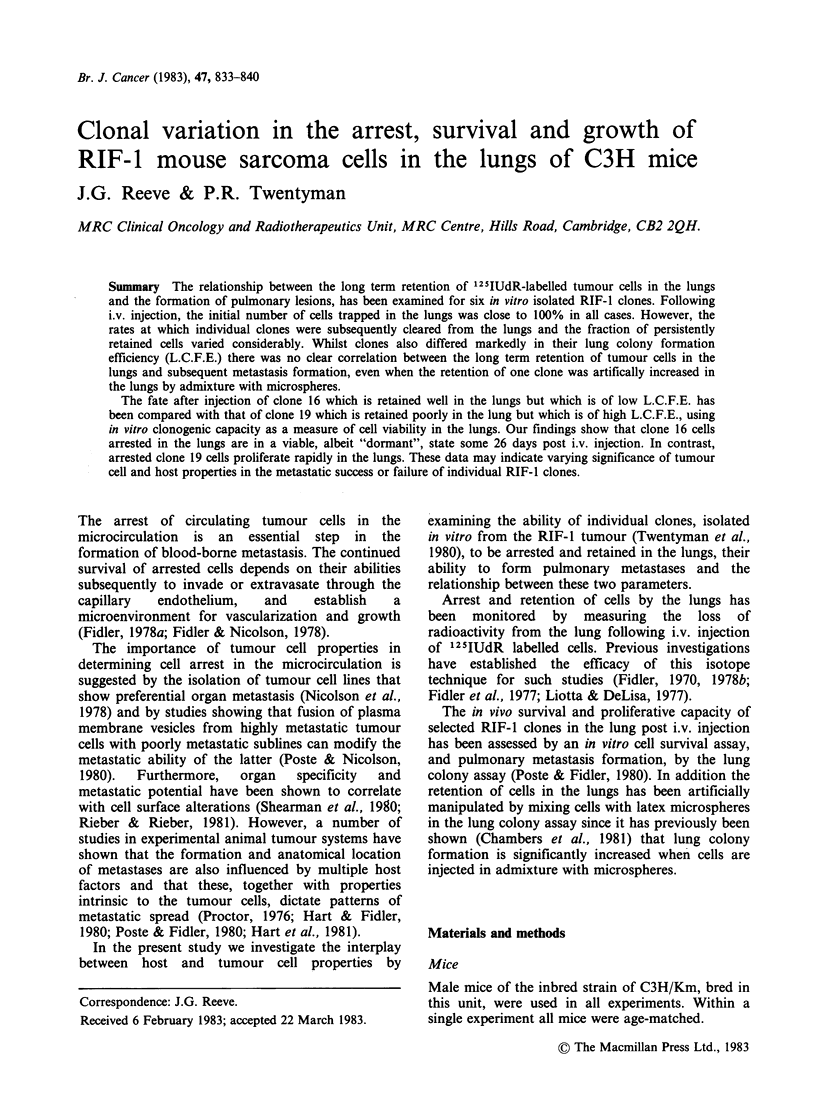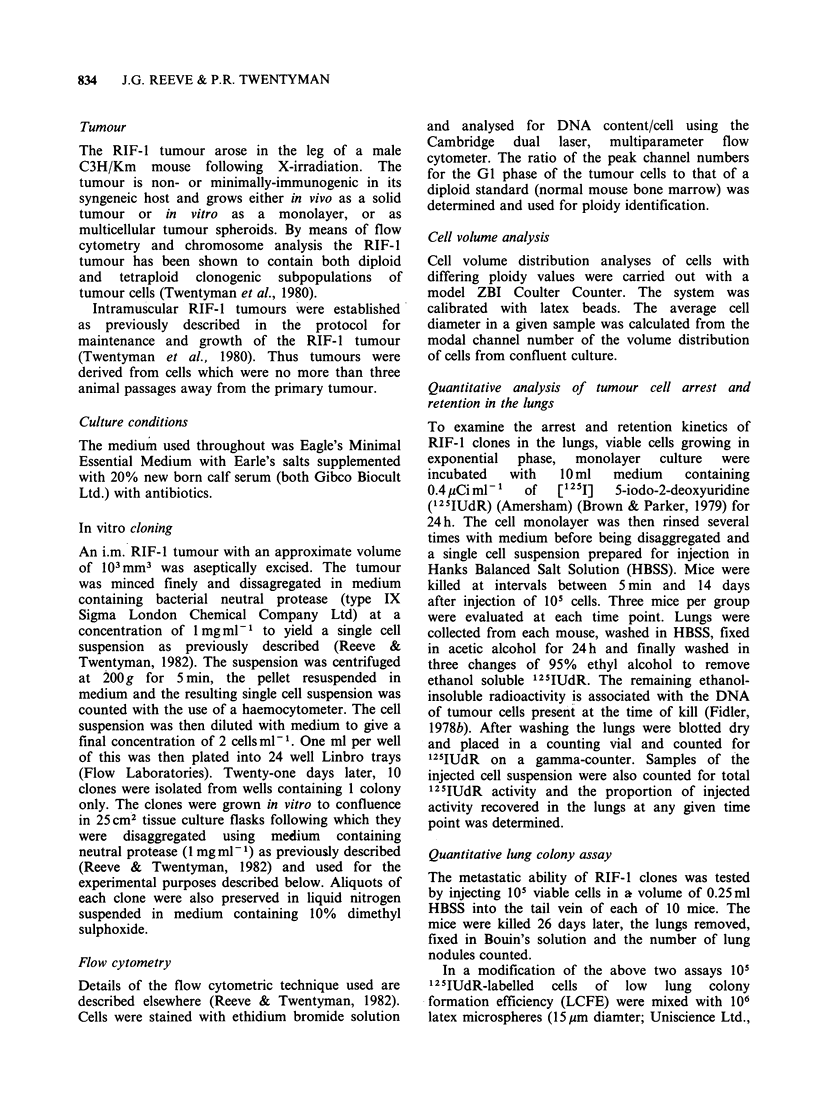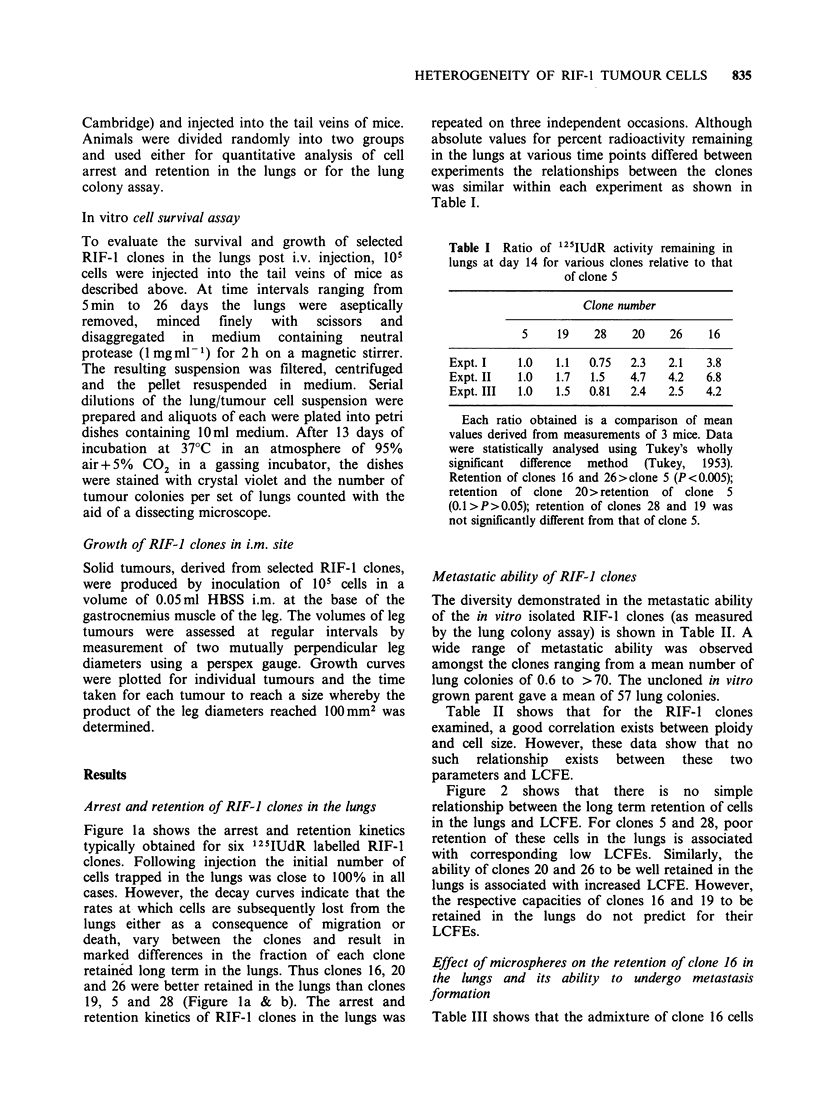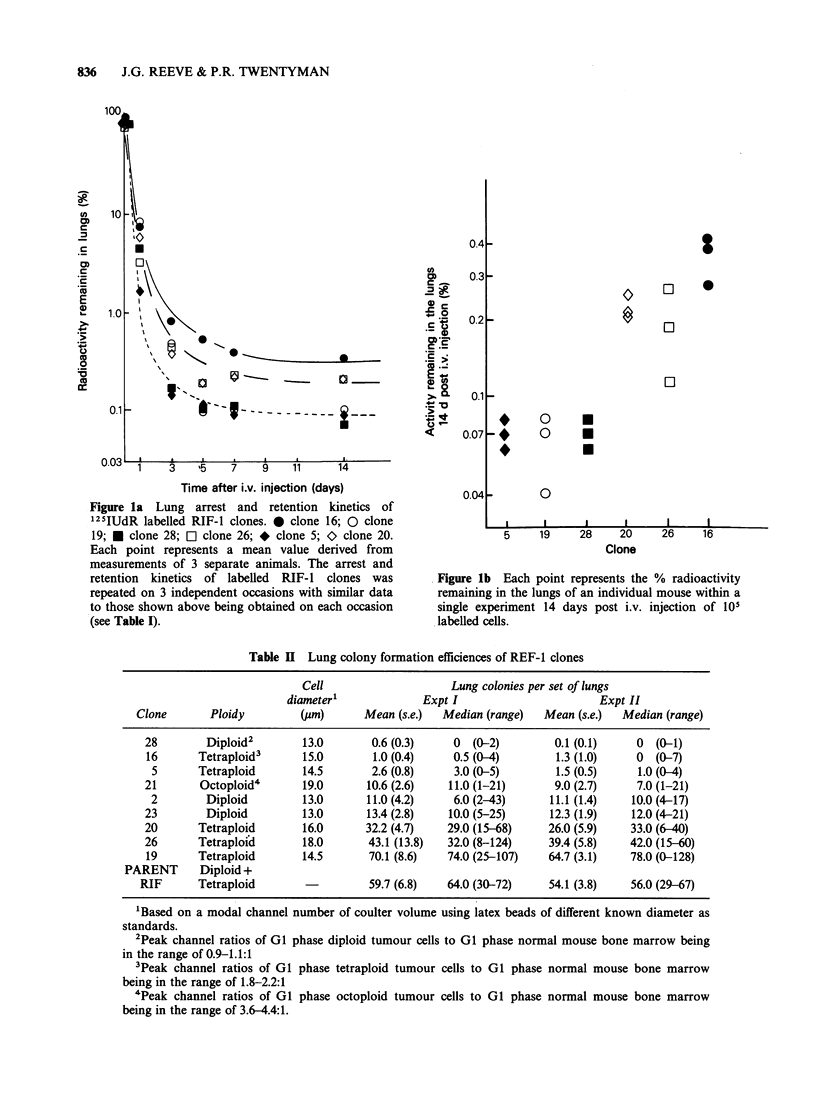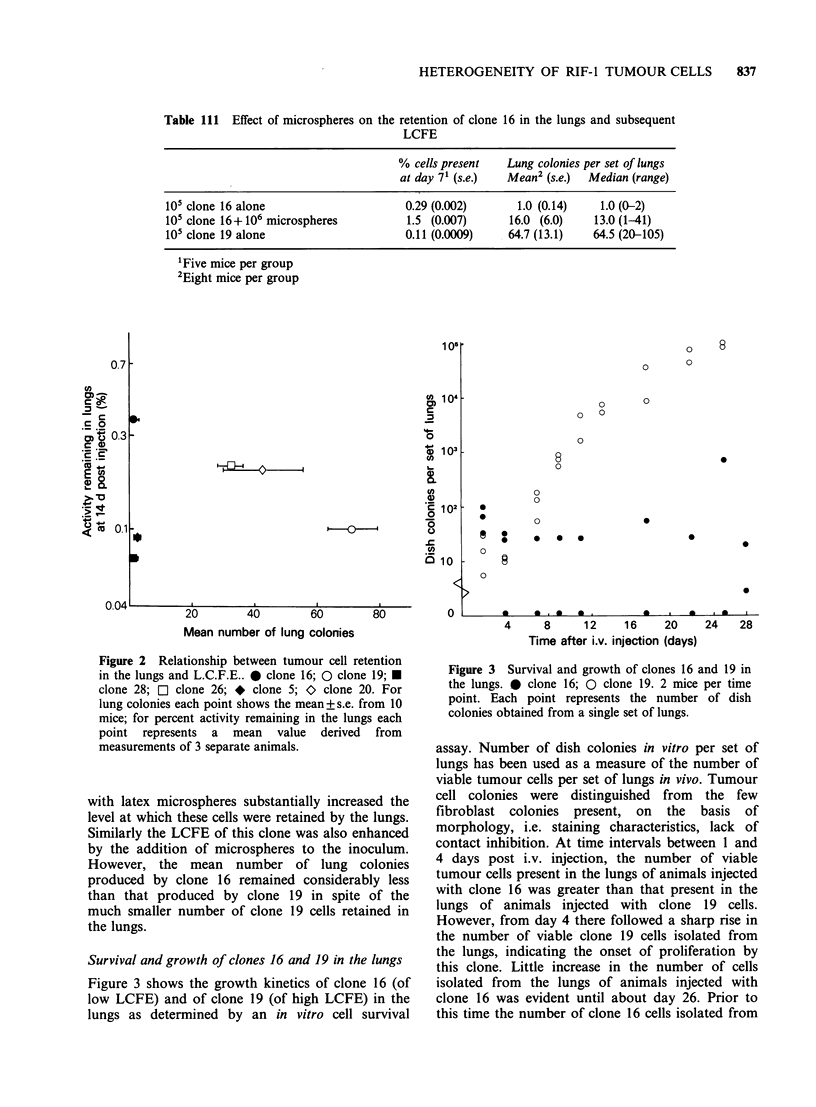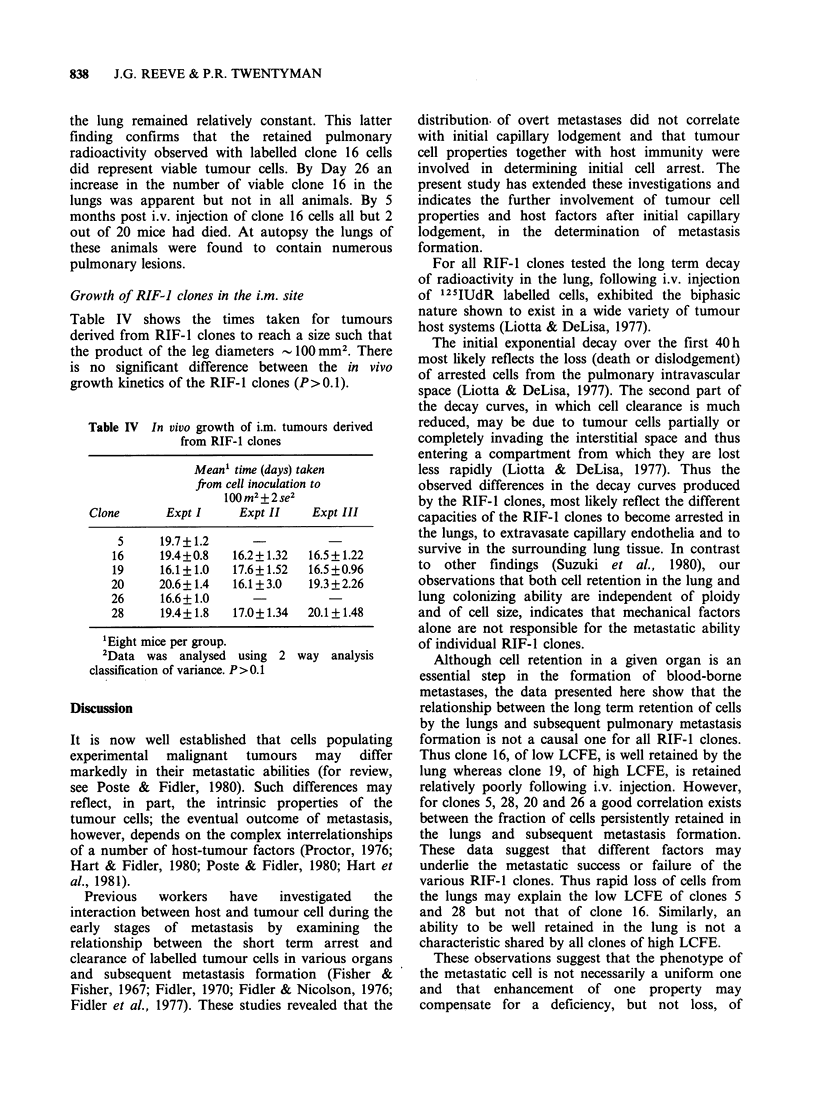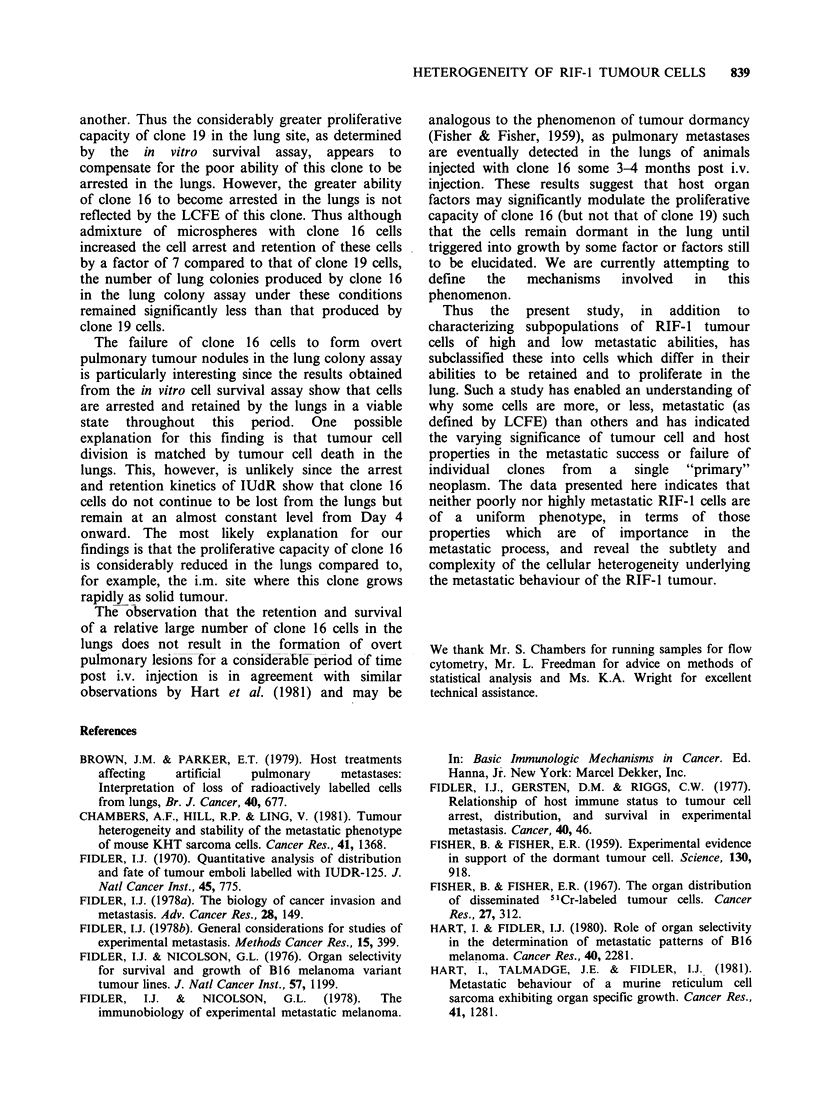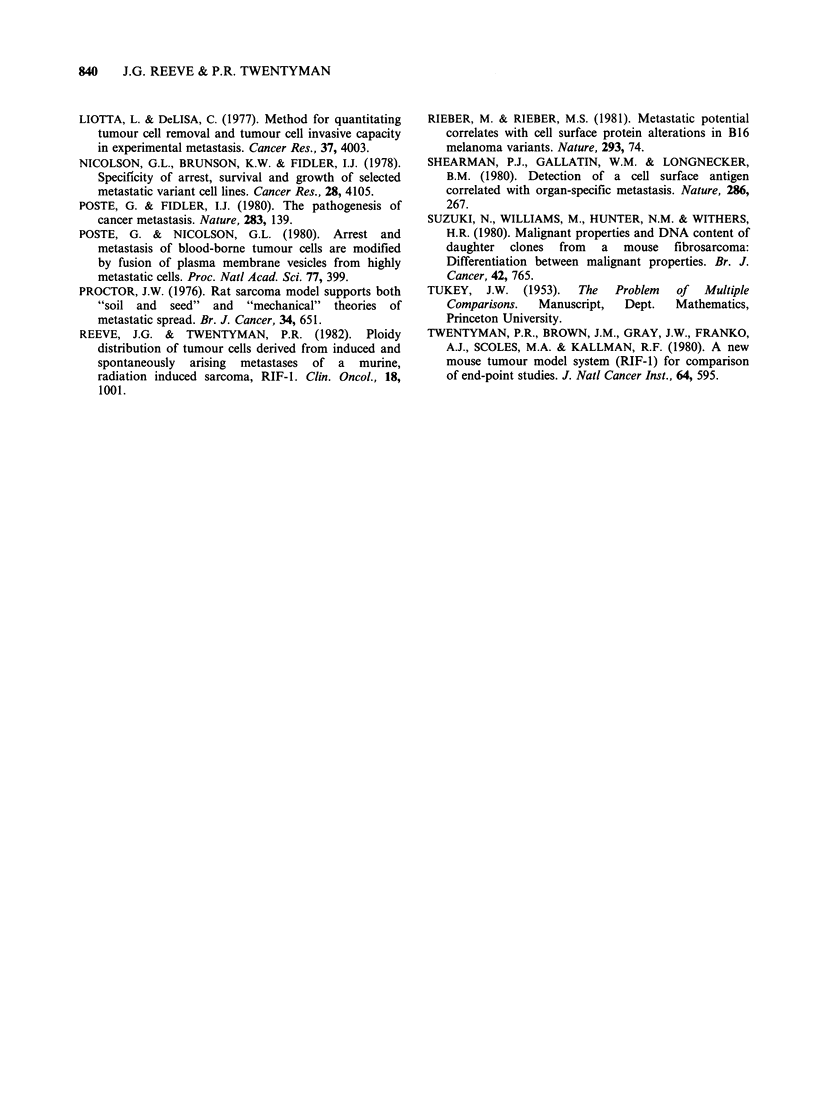# Clonal variation in the arrest, survival and growth of RIF-1 mouse sarcoma cells in the lungs of C3H mice.

**DOI:** 10.1038/bjc.1983.138

**Published:** 1983-06

**Authors:** J. G. Reeve, P. R. Twentyman

## Abstract

The relationship between the long term retention of 125IUdR-labelled tumour cells in the lungs and the formation of pulmonary lesions, has been examined for six in vitro isolated RIF-1 clones. Following i.v. injection, the initial number of cells trapped in the lungs was close to 100% in all cases. However, the rates at which individual clones were subsequently cleared from the lungs and the fraction of persistently retained cells varied considerably. Whilst clones also differed markedly in their lung colony formation efficiency (L.C.F.E.) there was no clear correlation between the long term retention of tumour cells in the lungs and subsequent metastasis formation, even when the retention of one clone was artificially increased in the lungs by admixture with microspheres. The fate after injection of clone 16 which is retained well in the lungs but which is of low L.C.F.E. has been compared with that of clone 19 which is retained poorly in the lung but which is of high L.C.F.E., using in vitro clonogenic capacity as a measure of cell viability in the lungs. Our findings show that clone 16 cells arrested in the lungs are in a viable, albeit "dormant", state some 26 days post i.v. injection. In contrast, arrested clone 19 cells proliferate rapidly in the lungs. These data may indicate varying significance of tumour cell and host properties in the metastatic success or failure of individual RIF-1 clones.


*Br. J. Cancer* (1983), **47**, 833–840

### Clonal variation in the arrest, survival and growth of RIF-1 mouse sarcoma cells in the lungs of C3H mice

J.G. Reeve & P.R. Twentyman

*MRC Clinical Oncology and Radiotherapeutics Unit, MRC Centre, Hills Road, Cambridge, CB2 2QH.*

**Summary** The relationship between the long term retention of $^{125}$IUdR-labelled tumour cells in the lungs and the formation of pulmonary lesions, has been examined for six *in vitro* isolated RIF-1 clones. Following i.v. injection, the initial number of cells trapped in the lungs was close to 100% in all cases. However, the rates at which individual clones were subsequently cleared from the lungs and the fraction of persistently retained cells varied considerably. Whilst clones also differed markedly in their lung colony formation efficiency (L.C.F.E.) there was no clear correlation between the long term retention of tumour cells in the lungs and subsequent metastasis formation, even when the retention of one clone was artifically increased in the lungs by admixture with microspheres.

The fate after injection of clone 16 which is retained well in the lungs but which is of low L.C.F.E. has been compared with that of clone 19 which is retained poorly in the lung but which is of high L.C.F.E., using *in vitro* clonogenic capacity as a measure of cell viability in the lungs. Our findings show that clone 16 cells arrested in the lungs are in a viable, albeit "dormant", state some 26 days post i.v. injection. In contrast, arrested clone 19 cells proliferate rapidly in the lungs. These data may indicate varying significance of tumour cell and host properties in the metastatic success or failure of individual RIF-1 clones.

The arrest of circulating tumour cells in the microcirculation is an essential step in the formation of blood-borne metastasis. The continued survival of arrested cells depends on their abilities subsequently to invade or extravasate through the capillary endothelium, and establish a microenvironment for vascularization and growth (Fidler, 1978*a*; Fidler & Nicolson, 1978).

The importance of tumour cell properties in determining cell arrest in the microcirculation is suggested by the isolation of tumour cell lines that show preferential organ metastasis (Nicolson *et al.*, 1978) and by studies showing that fusion of plasma membrane vesicles from highly metastatic tumour cells with poorly metastatic sublines can modify the metastatic ability of the latter (Poste & Nicolson, 1980). Furthermore, organ specificity and metastatic potential have been shown to correlate with cell surface alterations (Shearman *et al.*, 1980; Rieber & Rieber, 1981). However, a number of studies in experimental animal tumour systems have shown that the formation and anatomical location of metastases are also influenced by multiple host factors and that these, together with properties intrinsic to the tumour cells, dictate patterns of metastatic spread (Proctor, 1976; Hart & Fidler, 1980; Poste & Fidler, 1980; Hart *et al.*, 1981).

In the present study we investigate the interplay between host and tumour cell properties by examining the ability of individual clones, isolated *in vitro* from the RIF-1 tumour (Twentyman *et al.*, 1980), to be arrested and retained in the lungs, their ability to form pulmonary metastases and the relationship between these two parameters.

Arrest and retention of cells by the lungs has been monitored by measuring the loss of radioactivity from the lung following i.v. injection of $^{125}$IUdR labelled cells. Previous investigations have established the efficacy of this isotope technique for such studies (Fidler, 1970, 1978*b*; Fidler *et al.*, 1977; Liotta & DeLisa, 1977).

The *in vivo* survival and proliferative capacity of selected RIF-1 clones in the lung post i.v. injection has been assessed by an *in vitro* cell survival assay, and pulmonary metastasis formation, by the lung colony assay (Poste & Fidler, 1980). In addition the retention of cells in the lungs has been artificially manipulated by mixing cells with latex microspheres in the lung colony assay since it has previously been shown (Chambers *et al.*, 1981) that lung colony formation is significantly increased when cells are injected in admixture with microspheres.

Correspondence: J.G. Reeve.

Received 6 February 1983; accepted 22 March 1983.

### Materials and methods

### *Mice*

Male mice of the inbred strain of C3H/Km, bred in this unit, were used in all experiments. Within a single experiment all mice were age-matched.

© The Macmillan Press Ltd., 1983

*Tumour*

The RIF-1 tumour arose in the leg of a male C3H/Km mouse following X-irradiation. The tumour is non- or minimally-immunogenic in its syngeneic host and grows either *in vivo* as a solid tumour or *in vitro* as a monolayer, or as multicellular tumour spheroids. By means of flow cytometry and chromosome analysis the RIF-1 tumour has been shown to contain both diploid and tetraploid clonogenic subpopulations of tumour cells (Twentyman *et al.*, 1980).

Intramuscular RIF-1 tumours were established as previously described in the protocol for maintenance and growth of the RIF-1 tumour (Twentyman *et al.*, 1980). Thus tumours were derived from cells which were no more than three animal passages away from the primary tumour.

*Culture conditions*

The medium used throughout was Eagle's Minimal Essential Medium with Earle's salts supplemented with 20% new born calf serum (both Gibco Biocult Ltd.) with antibiotics.

In vitro *cloning*

An i.m. RIF-1 tumour with an approximate volume of $10^3$ mm$^3$ was aseptically excised. The tumour was minced finely and dissagregated in medium containing bacterial neutral protease (type IX Sigma London Chemical Company Ltd) at a concentration of 1 mg ml$^{-1}$ to yield a single cell suspension as previously described (Reeve & Twentyman, 1982). The suspension was centrifuged at 200 *g* for 5 min, the pellet resuspended in medium and the resulting single cell suspension was counted with the use of a haemocytometer. The cell suspension was then diluted with medium to give a final concentration of 2 cells ml$^{-1}$. One ml per well of this was then plated into 24 well Linbro trays (Flow Laboratories). Twenty-one days later, 10 clones were isolated from wells containing 1 colony only. The clones were grown *in vitro* to confluence in 25 cm$^2$ tissue culture flasks following which they were disaggregated using medium containing neutral protease (1 mg ml$^{-1}$) as previously described (Reeve & Twentyman, 1982) and used for the experimental purposes described below. Aliquots of each clone were also preserved in liquid nitrogen suspended in medium containing 10% dimethyl sulphoxide.

*Flow cytometry*

Details of the flow cytometric technique used are described elsewhere (Reeve & Twentyman, 1982). Cells were stained with ethidium bromide solution and analysed for DNA content/cell using the Cambridge dual laser, multiparameter flow cytometer. The ratio of the peak channel numbers for the G1 phase of the tumour cells to that of a diploid standard (normal mouse bone marrow) was determined and used for ploidy identification.

*Cell volume analysis*

Cell volume distribution analyses of cells with differing ploidy values were carried out with a model ZBI Coulter Counter. The system was calibrated with latex beads. The average cell diameter in a given sample was calculated from the modal channel number of the volume distribution of cells from confluent culture.

*Quantitative analysis of tumour cell arrest and retention in the lungs*

To examine the arrest and retention kinetics of RIF-1 clones in the lungs, viable cells growing in exponential phase, monolayer culture were incubated with 10 ml medium containing 0.4 $\mu$Ci ml$^{-1}$ of [$^{125}$I] 5-iodo-2-deoxyuridine ($^{125}$IUdR) (Amersham) (Brown & Parker, 1979) for 24 h. The cell monolayer was then rinsed several times with medium before being disaggregated and a single cell suspension prepared for injection in Hanks Balanced Salt Solution (HBSS). Mice were killed at intervals between 5 min and 14 days after injection of $10^5$ cells. Three mice per group were evaluated at each time point. Lungs were collected from each mouse, washed in HBSS, fixed in acetic alcohol for 24 h and finally washed in three changes of 95% ethyl alcohol to remove ethanol soluble $^{125}$IUdR. The remaining ethanol-insoluble radioactivity is associated with the DNA of tumour cells present at the time of kill (Fidler, 1978*b*). After washing the lungs were blotted dry and placed in a counting vial and counted for $^{125}$IUdR on a gamma-counter. Samples of the injected cell suspension were also counted for total $^{125}$IUdR activity and the proportion of injected activity recovered in the lungs at any given time point was determined.

*Quantitative lung colony assay*

The metastatic ability of RIF-1 clones was tested by injecting $10^5$ viable cells in a volume of 0.25 ml HBSS into the tail vein of each of 10 mice. The mice were killed 26 days later, the lungs removed, fixed in Bouin's solution and the number of lung nodules counted.

In a modification of the above two assays $10^5$ $^{125}$IUdR-labelled cells of low lung colony formation efficiency (LCFE) were mixed with $10^6$ latex microspheres (15 $\mu$m diamter; Uniscience Ltd.,

Cambridge) and injected into the tail veins of mice. Animals were divided randomly into two groups and used either for quantitative analysis of cell arrest and retention in the lungs or for the lung colony assay.

In vitro *cell survival assay*

To evaluate the survival and growth of selected RIF-1 clones in the lungs post i.v. injection, $10^5$ cells were injected into the tail veins of mice as described above. At time intervals ranging from 5 min to 26 days the lungs were aseptically removed, minced finely with scissors and disaggregated in medium containing neutral protease ($1\,mg\,ml^{-1}$) for 2 h on a magnetic stirrer. The resulting suspension was filtered, centrifuged and the pellet resuspended in medium. Serial dilutions of the lung/tumour cell suspension were prepared and aliquots of each were plated into petri dishes containing 10 ml medium. After 13 days of incubation at 37°C in an atmosphere of 95% air + 5% $CO_2$ in a gassing incubator, the dishes were stained with crystal violet and the number of tumour colonies per set of lungs counted with the aid of a dissecting microscope.

*Growth of RIF-1 clones in i.m. site*

Solid tumours, derived from selected RIF-1 clones, were produced by inoculation of $10^5$ cells in a volume of 0.05 ml HBSS i.m. at the base of the gastrocnemius muscle of the leg. The volumes of leg tumours were assessed at regular intervals by measurement of two mutually perpendicular leg diameters using a perspex gauge. Growth curves were plotted for individual tumours and the time taken for each tumour to reach a size whereby the product of the leg diameters reached $100\,mm^2$ was determined.

### Results

*Arrest and retention of RIF-1 clones in the lungs*

Figure 1a shows the arrest and retention kinetics typically obtained for six $^{125}$IUdR labelled RIF-1 clones. Following injection the initial number of cells trapped in the lungs was close to 100% in all cases. However, the decay curves indicate that the rates at which cells are subsequently lost from the lungs either as a consequence of migration or death, vary between the clones and result in marked differences in the fraction of each clone retained long term in the lungs. Thus clones 16, 20 and 26 were better retained in the lungs than clones 19, 5 and 28 (Figure 1a & b). The arrest and retention kinetics of RIF-1 clones in the lungs was repeated on three independent occasions. Although absolute values for percent radioactivity remaining in the lungs at various time points differed between experiments the relationships between the clones was similar within each experiment as shown in Table I.

**Table I** Ratio of $^{125}$IUdR activity remaining in lungs at day 14 for various clones relative to that of clone 5

| | *Clone number* | | | | | |
|---|---|---|---|---|---|---|
| | 5 | 19 | 28 | 20 | 26 | 16 |
| Expt. I | 1.0 | 1.1 | 0.75 | 2.3 | 2.1 | 3.8 |
| Expt. II | 1.0 | 1.7 | 1.5 | 4.7 | 4.2 | 6.8 |
| Expt. III | 1.0 | 1.5 | 0.81 | 2.4 | 2.5 | 4.2 |

Each ratio obtained is a comparison of mean values derived from measurements of 3 mice. Data were statistically analysed using Tukey's wholly significant difference method (Tukey, 1953). Retention of clones 16 and 26 > clone 5 ($P < 0.005$); retention of clone 20 > retention of clone 5 ($0.1 > P > 0.05$); retention of clones 28 and 19 was not significantly different from that of clone 5.

*Metastatic ability of RIF-1 clones*

The diversity demonstrated in the metastatic ability of the *in vitro* isolated RIF-1 clones (as measured by the lung colony assay) is shown in Table II. A wide range of metastatic ability was observed amongst the clones ranging from a mean number of lung colonies of 0.6 to > 70. The uncloned *in vitro* grown parent gave a mean of 57 lung colonies.

Table II shows that for the RIF-1 clones examined, a good correlation exists between ploidy and cell size. However, these data show that no such relationship exists between these two parameters and LCFE.

Figure 2 shows that there is no simple relationship between the long term retention of cells in the lungs and LCFE. For clones 5 and 28, poor retention of these cells in the lungs is associated with corresponding low LCFEs. Similarly, the ability of clones 20 and 26 to be well retained in the lungs is associated with increased LCFE. However, the respective capacities of clones 16 and 19 to be retained in the lungs do not predict for their LCFEs.

*Effect of microspheres on the retention of clone 16 in the lungs and its ability to undergo metastasis formation*

Table III shows that the admixture of clone 16 cells

**Figure 1a** Lung arrest and retention kinetics of $^{125}$IUdR labelled RIF-1 clones. ● clone 16; ○ clone 19; ■ clone 28; □ clone 26; ◆ clone 5; ◇ clone 20. Each point represents a mean value derived from measurements of 3 separate animals. The arrest and retention kinetics of labelled RIF-1 clones was repeated on 3 independent occasions with similar data to those shown above being obtained on each occasion (see **Table I**).

**Figure 1b** Each point represents the % radioactivity remaining in the lungs of an individual mouse within a single experiment 14 days post i.v. injection of $10^5$ labelled cells.

**Table II** Lung colony formation efficiences of REF-1 clones

| Clone | Ploidy | Cell diameter[1] (μm) | Lung colonies per set of lungs Expt I Mean (s.e.) | Expt I Median (range) | Expt II Mean (s.e.) | Expt II Median (range) |
|---|---|---|---|---|---|---|
| 28 | Diploid[2] | 13.0 | 0.6 (0.3) | 0 (0–2) | 0.1 (0.1) | 0 (0–1) |
| 16 | Tetraploid[3] | 15.0 | 1.0 (0.4) | 0.5 (0–4) | 1.3 (1.0) | 0 (0–7) |
| 5 | Tetraploid | 14.5 | 2.6 (0.8) | 3.0 (0–5) | 1.5 (0.5) | 1.0 (0–4) |
| 21 | Octoploid[4] | 19.0 | 10.6 (2.6) | 11.0 (1–21) | 9.0 (2.7) | 7.0 (1–21) |
| 2 | Diploid | 13.0 | 11.0 (4.2) | 6.0 (2–43) | 11.1 (1.4) | 10.0 (4–17) |
| 23 | Diploid | 13.0 | 13.4 (2.8) | 10.0 (5–25) | 12.3 (1.9) | 12.0 (4–21) |
| 20 | Tetraploid | 16.0 | 32.2 (4.7) | 29.0 (15–68) | 26.0 (5.9) | 33.0 (6–40) |
| 26 | Tetraploid | 18.0 | 43.1 (13.8) | 32.0 (8–124) | 39.4 (5.8) | 42.0 (15–60) |
| 19 | Tetraploid | 14.5 | 70.1 (8.6) | 74.0 (25–107) | 64.7 (3.1) | 78.0 (0–128) |
| PARENT RIF | Diploid + Tetraploid | — | 59.7 (6.8) | 64.0 (30–72) | 54.1 (3.8) | 56.0 (29–67) |

[1]Based on a modal channel number of coulter volume using latex beads of different known diameter as standards.

[2]Peak channel ratios of G1 phase diploid tumour cells to G1 phase normal mouse bone marrow being in the range of 0.9–1.1:1

[3]Peak channel ratios of G1 phase tetraploid tumour cells to G1 phase normal mouse bone marrow being in the range of 1.8–2.2:1

[4]Peak channel ratios of G1 phase octoploid tumour cells to G1 phase normal mouse bone marrow being in the range of 3.6–4.4:1.

**Table 111** Effect of microspheres on the retention of clone 16 in the lungs and subsequent LCFE

| | *% cells present at day 7*[1] *(s.e.)* | *Lung colonies per set of lungs Mean*[2] *(s.e.)* | *Median (range)* |
|---|---|---|---|
| $10^5$ clone 16 alone | 0.29 (0.002) | 1.0 (0.14) | 1.0 (0–2) |
| $10^5$ clone 16 + $10^6$ microspheres | 1.5 (0.007) | 16.0 (6.0) | 13.0 (1–41) |
| $10^5$ clone 19 alone | 0.11 (0.0009) | 64.7 (13.1) | 64.5 (20–105) |

[1]Five mice per group
[2]Eight mice per group

**Figure 2** Relationship between tumour cell retention in the lungs and L.C.F.E.. ● clone 16; ○ clone 19; ■ clone 28; □ clone 26; ◆ clone 5; ◇ clone 20. For lung colonies each point shows the mean±s.e. from 10 mice; for percent activity remaining in the lungs each point represents a mean value derived from measurements of 3 separate animals.

**Figure 3** Survival and growth of clones 16 and 19 in the lungs. ● clone 16; ○ clone 19. 2 mice per time point. Each point represents the number of dish colonies obtained from a single set of lungs.

with latex microspheres substantially increased the level at which these cells were retained by the lungs. Similarly the LCFE of this clone was also enhanced by the addition of microspheres to the inoculum. However, the mean number of lung colonies produced by clone 16 remained considerably less than that produced by clone 19 in spite of the much smaller number of clone 19 cells retained in the lungs.

*Survival and growth of clones 16 and 19 in the lungs*

Figure 3 shows the growth kinetics of clone 16 (of low LCFE) and of clone 19 (of high LCFE) in the lungs as determined by an *in vitro* cell survival assay. Number of dish colonies *in vitro* per set of lungs has been used as a measure of the number of viable tumour cells per set of lungs *in vivo*. Tumour cell colonies were distinguished from the few fibroblast colonies present, on the basis of morphology, i.e. staining characteristics, lack of contact inhibition. At time intervals between 1 and 4 days post i.v. injection, the number of viable tumour cells present in the lungs of animals injected with clone 16 was greater than that present in the lungs of animals injected with clone 19 cells. However, from day 4 there followed a sharp rise in the number of viable clone 19 cells isolated from the lungs, indicating the onset of proliferation by this clone. Little increase in the number of cells isolated from the lungs of animals injected with clone 16 was evident until about day 26. Prior to this time the number of clone 16 cells isolated from the lung remained relatively constant. This latter finding confirms that the retained pulmonary radioactivity observed with labelled clone 16 cells did represent viable tumour cells. By Day 26 an increase in the number of viable clone 16 in the lungs was apparent but not in all animals. By 5 months post i.v. injection of clone 16 cells all but 2 out of 20 mice had died. At autopsy the lungs of these animals were found to contain numerous pulmonary lesions.

*Growth of RIF-1 clones in the i.m. site*

Table IV shows the times taken for tumours derived from RIF-1 clones to reach a size such that the product of the leg diameters $\sim 100\,mm^2$. There is no significant difference between the *in vivo* growth kinetics of the RIF-1 clones ($P > 0.1$).

**Table IV** *In vivo* growth of i.m. tumours derived from RIF-1 clones

| | *Mean*[1] *time (days) taken from cell inoculation to* $100\,m^2 \pm 2\,se^2$ | | |
|---|---|---|---|
| *Clone* | *Expt I* | *Expt II* | *Expt III* |
| 5 | 19.7±1.2 | — | — |
| 16 | 19.4±0.8 | 16.2±1.32 | 16.5±1.22 |
| 19 | 16.1±1.0 | 17.6±1.52 | 16.5±0.96 |
| 20 | 20.6±1.4 | 16.1±3.0 | 19.3±2.26 |
| 26 | 16.6±1.0 | — | — |
| 28 | 19.4±1.8 | 17.0±1.34 | 20.1±1.48 |

[1]Eight mice per group.
[2]Data was analysed using 2 way analysis classification of variance. $P > 0.1$

### Discussion

It is now well established that cells populating experimental malignant tumours may differ markedly in their metastatic abilities (for review, see Poste & Fidler, 1980). Such differences may reflect, in part, the intrinsic properties of the tumour cells; the eventual outcome of metastasis, however, depends on the complex interrelationships of a number of host-tumour factors (Proctor, 1976; Hart & Fidler, 1980; Poste & Fidler, 1980; Hart *et al*., 1981).

Previous workers have investigated the interaction between host and tumour cell during the early stages of metastasis by examining the relationship between the short term arrest and clearance of labelled tumour cells in various organs and subsequent metastasis formation (Fisher & Fisher, 1967; Fidler, 1970; Fidler & Nicolson, 1976; Fidler *et al.,* 1977). These studies revealed that the distribution of overt metastases did not correlate with initial capillary lodgement and that tumour cell properties together with host immunity were involved in determining initial cell arrest. The present study has extended these investigations and indicates the further involvement of tumour cell properties and host factors after initial capillary lodgement, in the determination of metastasis formation.

For all RIF-1 clones tested the long term decay of radioactivity in the lung, following i.v. injection of $^{125}$IUdR labelled cells, exhibited the biphasic nature shown to exist in a wide variety of tumour host systems (Liotta & DeLisa, 1977).

The initial exponential decay over the first 40 h most likely reflects the loss (death or dislodgement) of arrested cells from the pulmonary intravascular space (Liotta & DeLisa, 1977). The second part of the decay curves, in which cell clearance is much reduced, may be due to tumour cells partially or completely invading the interstitial space and thus entering a compartment from which they are lost less rapidly (Liotta & DeLisa, 1977). Thus the observed differences in the decay curves produced by the RIF-1 clones, most likely reflect the different capacities of the RIF-1 clones to become arrested in the lungs, to extravasate capillary endothelia and to survive in the surrounding lung tissue. In contrast to other findings (Suzuki *et al.,* 1980), our observations that both cell retention in the lung and lung colonizing ability are independent of ploidy and of cell size, indicates that mechanical factors alone are not responsible for the metastatic ability of individual RIF-1 clones.

Although cell retention in a given organ is an essential step in the formation of blood-borne metastases, the data presented here show that the relationship between the long term retention of cells by the lungs and subsequent pulmonary metastasis formation is not a causal one for all RIF-1 clones. Thus clone 16, of low LCFE, is well retained by the lung whereas clone 19, of high LCFE, is retained relatively poorly following i.v. injection. However, for clones 5, 28, 20 and 26 a good correlation exists between the fraction of cells persistently retained in the lungs and subsequent metastasis formation. These data suggest that different factors may underlie the metastatic success or failure of the various RIF-1 clones. Thus rapid loss of cells from the lungs may explain the low LCFE of clones 5 and 28 but not that of clone 16. Similarly, an ability to be well retained in the lung is not a characteristic shared by all clones of high LCFE.

These observations suggest that the phenotype of the metastatic cell is not necessarily a uniform one and that enhancement of one property may compensate for a deficiency, but not loss, of another. Thus the considerably greater proliferative capacity of clone 19 in the lung site, as determined by the *in vitro* survival assay, appears to compensate for the poor ability of this clone to be arrested in the lungs. However, the greater ability of clone 16 to become arrested in the lungs is not reflected by the LCFE of this clone. Thus although admixture of microspheres with clone 16 cells increased the cell arrest and retention of these cells by a factor of 7 compared to that of clone 19 cells, the number of lung colonies produced by clone 16 in the lung colony assay under these conditions remained significantly less than that produced by clone 19 cells.

The failure of clone 16 cells to form overt pulmonary tumour nodules in the lung colony assay is particularly interesting since the results obtained from the *in vitro* cell survival assay show that cells are arrested and retained by the lungs in a viable state throughout this period. One possible explanation for this finding is that tumour cell division is matched by tumour cell death in the lungs. This, however, is unlikely since the arrest and retention kinetics of IUdR show that clone 16 cells do not continue to be lost from the lungs but remain at an almost constant level from Day 4 onward. The most likely explanation for our findings is that the proliferative capacity of clone 16 is considerably reduced in the lungs compared to, for example, the i.m. site where this clone grows rapidly as solid tumour.

The observation that the retention and survival of a relative large number of clone 16 cells in the lungs does not result in the formation of overt pulmonary lesions for a considerable period of time post i.v. injection is in agreement with similar observations by Hart *et al.* (1981) and may be analogous to the phenomenon of tumour dormancy (Fisher & Fisher, 1959), as pulmonary metastases are eventually detected in the lungs of animals injected with clone 16 some 3–4 months post i.v. injection. These results suggest that host organ factors may significantly modulate the proliferative capacity of clone 16 (but not that of clone 19) such that the cells remain dormant in the lung until triggered into growth by some factor or factors still to be elucidated. We are currently attempting to define the mechanisms involved in this phenomenon.

Thus the present study, in addition to characterizing subpopulations of RIF-1 tumour cells of high and low metastatic abilities, has subclassified these into cells which differ in their abilities to be retained and to proliferate in the lung. Such a study has enabled an understanding of why some cells are more, or less, metastatic (as defined by LCFE) than others and has indicated the varying significance of tumour cell and host properties in the metastatic success or failure of individual clones from a single "primary" neoplasm. The data presented here indicates that neither poorly nor highly metastatic RIF-1 cells are of a uniform phenotype, in terms of those properties which are of importance in the metastatic process, and reveal the subtlety and complexity of the cellular heterogeneity underlying the metastatic behaviour of the RIF-1 tumour.

We thank Mr. S. Chambers for running samples for flow cytometry, Mr. L. Freedman for advice on methods of statistical analysis and Ms. K.A. Wright for excellent technical assistance.

### References

BROWN, J.M. & PARKER, E.T. (1979). Host treatments affecting artificial pulmonary metastases: Interpretation of loss of radioactively labelled cells from lungs, *Br. J. Cancer*, **40**, 677.

CHAMBERS, A.F., HILL, R.P. & LING, V. (1981). Tumour heterogeneity and stability of the metastatic phenotype of mouse KHT sarcoma cells. *Cancer Res.*, **41**, 1368.

FIDLER, I.J. (1970). Quantitative analysis of distribution and fate of tumour emboli labelled with IUDR-125. *J. Natl Cancer Inst.*, **45**, 775.

FIDLER, I.J. (1978*a*). The biology of cancer invasion and metastasis. *Adv. Cancer Res.*, **28**, 149.

FIDLER, I.J. (1978*b*). General considerations for studies of experimental metastasis. *Methods Cancer Res.*, **15**, 399.

FIDLER, I.J. & NICOLSON, G.L. (1976). Organ selectivity for survival and growth of B16 melanoma variant tumour lines. *J. Natl Cancer Inst.*, **57**, 1199.

FIDLER, I.J. & NICOLSON, G.L. (1978). The immunobiology of experimental metastatic melanoma. In: *Basic Immunologic Mechanisms in Cancer*. Ed. Hanna, Jr. New York: Marcel Dekker, Inc.

FIDLER, I.J., GERSTEN, D.M. & RIGGS, C.W. (1977). Relationship of host immune status to tumour cell arrest, distribution, and survival in experimental metastasis. *Cancer*, **40**, 46.

FISHER, B. & FISHER, E.R. (1959). Experimental evidence in support of the dormant tumour cell. *Science*, **130**, 918.

FISHER, B. & FISHER, E.R. (1967). The organ distribution of disseminated $^{51}$Cr-labeled tumour cells. *Cancer Res.*, **27**, 312.

HART, I. & FIDLER, I.J. (1980). Role of organ selectivity in the determination of metastatic patterns of B16 melanoma. *Cancer Res.*, **40**, 2281.

HART, I., TALMADGE, J.E. & FIDLER, I.J. (1981). Metastatic behaviour of a murine reticulum cell sarcoma exhibiting organ specific growth. *Cancer Res.*, **41**, 1281.

LIOTTA, L. & DeLISA, C. (1977). Method for quantitating tumour cell removal and tumour cell invasive capacity in experimental metastasis. *Cancer Res.*, **37**, 4003.

NICOLSON, G.L., BRUNSON, K.W. & FIDLER, I.J. (1978). Specificity of arrest, survival and growth of selected metastatic variant cell lines. *Cancer Res.*, **28**, 4105.

POSTE, G. & FIDLER, I.J. (1980). The pathogenesis of cancer metastasis. *Nature*, **283**, 139.

POSTE, G. & NICOLSON, G.L. (1980). Arrest and metastasis of blood-borne tumour cells are modified by fusion of plasma membrane vesicles from highly metastatic cells. *Proc. Natl Acad. Sci.* **77**, 399.

PROCTOR, J.W. (1976). Rat sarcoma model supports both "soil and seed" and "mechanical" theories of metastatic spread. *Br. J. Cancer*, **34**, 651.

REEVE, J.G. & TWENTYMAN, P.R. (1982). Ploidy distribution of tumour cells derived from induced and spontaneously arising metastases of a murine, radiation induced sarcoma, RIF-1. *Clin. Oncol.*, **18**, 1001.

RIEBER, M. & RIEBER, M.S. (1981). Metastatic potential correlates with cell surface protein alterations in B16 melanoma variants. *Nature*, **293**, 74.

SHEARMAN, P.J., GALLATIN, W.M. & LONGNECKER, B.M. (1980). Detection of a cell surface antigen correlated with organ-specific metastasis. *Nature*, **286**, 267.

SUZUKI, N., WILLIAMS, M., HUNTER, N.M. & WITHERS, H.R. (1980). Malignant properties and DNA content of daughter clones from a mouse fibrosarcoma: Differentiation between malignant properties. *Br. J. Cancer*, **42**, 765.

TUKEY, J.W. (1953). *The Problem of Multiple Comparisons*. Manuscript, Dept. Mathematics, Princeton University.

TWENTYMAN, P.R., BROWN, J.M., GRAY, J.W., FRANKO, A.J., SCOLES, M.A. & KALLMAN, R.F. (1980). A new mouse tumour model system (RIF-1) for comparison of end-point studies. *J. Natl Cancer Inst.*, **64**, 595.